# Role of Salivary Biomarkers in Detection of Cardiovascular Diseases (CVD)

**DOI:** 10.3390/proteomes5030021

**Published:** 2017-08-07

**Authors:** Saad Abdul Rehman, Zohaib Khurshid, Fayez Hussain Niazi, Mustafa Naseem, Hamed Al Waddani, Haafsa Arshad Sahibzada, Rabia Sannam Khan

**Affiliations:** 1 Department of Orthodontics, College of Dentistry, Karachi Medical and Dental College, Karachi 74700, Pakistan; saadmemon_786@hotmail.com; 2 Department of Prosthodontics and Implantology, College of Dentistry, King Faisal University, Al-Ahsa 31982, Saudi Arabia; hwadani@hotmail.com; 3 Department of Restorative Dentistry, Dar Al Uloom University, Riyadh 13314, Saudia Arabia; fayez.h@dau.edu.sa; 4 Department of Preventive Dental Sciences, Dar Al Uloom University, Riyadh 13314, Saudia Arabia; m.naseem@dau.edu.sa; 5 Department of Oral Medicine, Islamabad Dental Hospital (IDH), Islamabad 44000, Pakistan; Haafsa.OralMed16@iideas.edu.pk; 6 Department of Oral Pathology, College of Dentistry, Baqai University, Karachi 74600, Pakistan; rabia.sannam.khan@gmail.com

**Keywords:** saliva, proteins, biomarkers, cardiovascular diseases and diagnosis

## Abstract

Human whole mouth saliva (WMS) is secreted by salivary glands, namely parotid, submandibular/sublingual and other minor glands of the oral cavity. It is secreted in a systematic way, and contain informative proteins and peptides for the early detection of contagious diseases and organ-related diseases. The role of WMS as a liquid biopsy for the detection of cardiovascular diseases (CVD) through Myoglobin (MYO), Cardiac troponin I (cTnI), Creatine phosphokinase MB (CK-MB), Myeloperoxidase (MPO), brain natriuretic peptide (NT-proBNP), Exosomal miRNA, C-Reactive Protein (CRP), Matrix metalloproteinase-8 (MMP-8), MMP-9 and tissue inhibitor of MMP-8 (TIMP-1), leukotriene B4 has been well reported in last decade, that have been reviewed in the literature comprehensively below.

## 1. Introduction

Nearly 31% of deaths globally are reported to be caused by cardiovascular diseases (CVDs), which is recognized to be one of the leading causes of death annually by the World Health Organization (WHO) [1]. Multiple diseases are included in CVDs [2], but the most vicious is acute myocardial infarction (AMI). AMI is a life-threatening complication, and is one of the most frequent causes of death [1,3]. Currently, the diagnosis of CVDs is based on subjective and objective clinical findings, electrocardiogram and time-significant multiple serum biomarkers [4,5]. Despite improvements in medicine, acute myocardial infarction remains one of the leading causes of mortality and morbidity. The pathological disease occurs because of ischemia of the cardiac vasculature leading to necrosis of the area involved [6]. The disease accounts for nearly 50% of all CVDs that occur worldwide [3].

**Figure 1:**
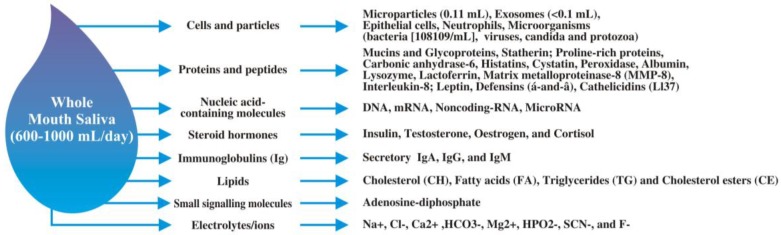
Illustration of a drop of whole mouth saliva representing its detailed composition.

Saliva, a known biofluid, is also known to be a plasma ultrafiltrate [5,7]. Currently, nearly 1000 different proteins and 19,000 unique peptide sequences have been detected in saliva [8]. Whole mouth saliva (WMS) is a mixture of different secretions that are produced by the major and minor salivary glands, gingival crevicular fluid (GCF), mucosal transudations, serum and blood sheds from oral wounds, desquamated epithelial cells, acquired pellicles, bacterial products, viruses and fungi, other cellular components and food debris [8,9,10,11,12]. Figure 1 illustrates the detailed composition of WMS. Its collection was reported by our group in a comprehensive review paper on human saliva collection via advanced devices manufactured by Salimetrics^®^ (State College, PA, USA), DNAGenotek (Kanata, ON, Canada), Oasis Diagnostics^®^ Corporation (Vancouver, WA, USA). These devices bring about a revolution in collection methods, transportation and screening of epidemic areas, which they do easily without any contamination [13].

**Figure 2:**
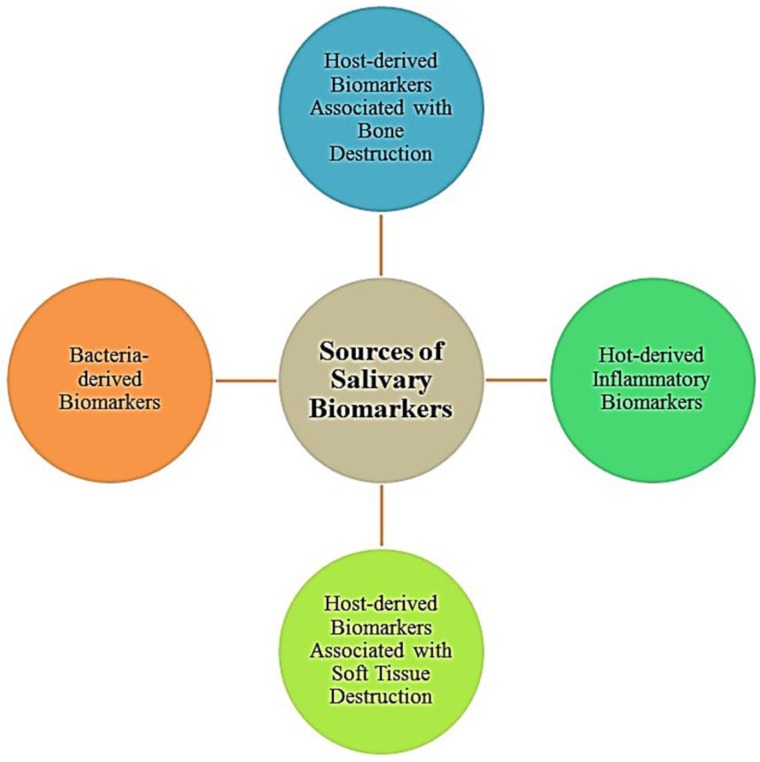
Illustration is representing the sources of human salivary biomarkers.

Over the last decade, human saliva has attracted attention as a liquid biopsy for the detection of oral diseases like dental caries, gingivitis, periodontitis (chronic/aggressive), Bechet disease, oral squamous cell carcinoma, cleft palate and lips, salivary gland diseases, oral leukoplakia, chronic graft-versus-host disease (cGVHD), and systematic diseases such as breast cancer, diabetes, human immune deficiency virus (HIV). Biomarkers are defined as a biological molecules found in blood, saliva and other body fluids, or tissues that are a sign of a normal or abnormal process, or of a condition or disease. These biomarkers are classified as strong (S), questionable (Q) and potential (P). In Figure 2, we illustrate the sources of human salivary biomarkers on the oral cavity. They represent the pathological or physiological changes that are occurring in the human body. Even though the majority of the biomarkers are tested and found positive in the serum, not all of them can detected via blood. To date, only a handful of studies have demonstrated a relationship between the serum and the salivary levels of these biomarkers.

Many of these biomarkers enter saliva through blood via passive diffusion, active transport or extracellular ultra-filtration. Therefore, saliva can be a good reflection of the physiological function of the body [4]. Scientists are working on salivary biomarkers around the world, in order to indicate, estimate, prognosis and diagnose various conditions. They would serve as a non-invasive, inherently painless, rapidly collected method, easily and economically performed by minimally trained personnel. Advancements in “*omics*” sciences toward salivary research will aid in identification of biomarkers related to healthy and diseased state [14].

## 2. Salivary Biomarkers in Cardiovascular Disease (CVD) Detection

Literature has been published on the importance of salivary biomarkers in the diagnosis of CVDs, which includes Myoglobin (MYO), Cardiac troponin I (cTnI), Creatine phosphokinase MB (CK-MB), Myeloperoxidase (MPO), brain natriuretic peptide (NT-proBNP), Exosomal miRNA, C-Reactive Protein (CRP), Matrix metalloproteinase-8 (MMP-8), MMP-9 and tissue inhibitor of MMP-8 (TIMP-1) [1,2,3,15,16,17]. Myoglobin, which appears in both serum and saliva bio-fluids, can be used to detect AMI. Miller and his coworkers conducted research, and established that salivary myoglobin levels were greater within 48 h of the onset of angina in AMI patients [4]. It was proved that the unstimulated saliva concentration of Cardiac troponin-I (cTnI) at the onset of 12 h and 24 h of Acute Myocardial Infection and creatine phosphokinase-MB (CK-MB) increased in patients with AMI compared to Non-AMI controls. This study also proved the strong link between levels of serum and salivary CK-MB and CPK, indicating that saliva-based tests may provide an easy and convenient way of providing point-of-care testing of CVDs [3]. CRP is one of the inflammatory mediators of our body that is produced to trigger the complement cascade in response to acute injuries and infections. It also contributes and plays a vital role in atherogenesis [4]. Studies proving a group of salivary biomarkers including CRP, MYO, and MPO as a diagnostic tool for AMI have shown a sensitivity of 90–100% [5,18], but BNP salivary levels, in contrast to serum BNP, were lower than the mean value of the control group [3]. Many inflammatory components and adhesion molecules have also been identified as a salivary biomarker for the diagnosis of AMI, including IL-6, MMP-9, soluble intercellular adhesion molecule (sICAM-1), a soluble form of CD40 ligand (sCD40-L), CRP, cTnI and adiponectin [16,17]. Matrix metalloproteinase-8 has a well-established role in the repair and remodeling process of myocardial tissue after damage. Tissue inhibitor TIMP-1 inhibits MMP-8. So the ratio of MMP-8/TIMP-1 also projects the acceptable image of progression and severity of CVDs [1]. Miller et al. elaborated that the concentrations of CRP, brain natriuretic peptide (NT-proBNP), matrix metallopeptidase 9 and tumor necrosis factor α in saliva were greater than in the AMI patients [7].

Some research has been conducted to test and check the compatibility of salivary biomarkers with serum biomarkers [4,7]. Labat et al. investigated and found that there is a strong, positive and significant correlation between salivary and serum CRP levels among patients with ischemic heart disease (IHD). Through the collection of saliva and plasma samples from 250 individuals with a prior history of cardiovascular disease, salivary levels of CRP, prostaglandin E2 (PGE2), leukotriene B4 (LTB4), matrix metaaloproteinase 9 (MMP9), creatinine and lysozyme were measured, with the results indicating that saliva could be an alternative means for evaluation of cardiovascular risk [18]. This indicates that CRP can be used to detect and monitor CVDs [2]. Kossaify et al. studied certain specific cardiac biomarkers and a few non-specific inflammatory biomarkers that have significant roles in inflammation and plaque instability. They elaborated that levels of CRP, CK-MB, cardiac troponin I, cardiac troponin T, some interleukins (IL), tumor necrosis factor alpha (TNF-Alpha), MMPs, and MPO are associated with both saliva and serum [3]. The conditions leading to increase or alteration in the levels of these biomarkers specifically related to cardiac tissues include myocardial injury, myocardial inflammation and myocardial stress. Other general conditions include neuroendocrine activation, plaque instability, atherosclerotic processes, platelet activation, endothelial dysfunction and oxidative stresses. Moreover, Thul et al. collected saliva from 254 individuals and took the ultrasound measurements of the thickness of the carotid artery’s intima media layer and analyzed the lipid mediator resolvin D1 by means of an enzyme-linked immunosorbent assay, and checked the association between leukotriene B4 and resolvin D1. Results depicted the independent prediction of intima media thickness, and both served as a biomarker of non-resolving inflammation [19]. In addition to this, the presence and activity of inflammatory mediators in saliva suggests its constant low level in the oral cavity of healthy individuals in association with age [20].

Folley et al. also assessed the utility of unstimulated whole saliva to determine cardiovascular health. Unstimulated whole saliva and serum was collected from 29 patients with CVD, at intervals of 0.8, 16, 24 and 48 h after invasive cardiac procedures, by using gingival and sublingual swabs. Results showed that the oral fluid reflected most of the biomarkers present in serum, which ultimately suggests that saliva serves as an essential tool for assessing cardiac ischemia or necrosis [21]. Rahim et al. also reviewed the biomarkers in salivary proteome to predict acute myocardial infarction [22]. A detailed description of identified salivary biomarkers for the detection of CVD biomarkers is presented in Table 1 below.

## 3. Future Prospects

The potentiality of saliva for liquid biopsy has been reported vastly as a major diagnostic tool for medical conditions and dental diseases. The non-invasive nature and easy sampling with information related to body health makes it attractive for clinical and private practice. Few markers mentioned above are general and non-specific, so a panel of more specific markers will be required to make it an acceptable diagnostic fluid for CVD. Recent introdcution of programmable bio-nano-chip (P-BNC) system contributes as a revolution in the salivary diagnostic technology toward the CVD detection. Other biosensor systems, cardio Micro-Electro-Mechanical Systems (MEMS) are also available, and with the help of latest lab on a chip system, these will create breakthroughs in hospital practice and general well-being of human health [25]. Future developments in this diagnostic tool will lead to further advancement in certain devices that can modify the approach towards screening a wide range of cardiovascular diseases (CVD).

## Figures and Tables

**Table 1:** Representation of salivary biomarkers studies for the detection of cardiovascular diseases (CVD).

Author Name and Year	Sample Type	Disease Conditions	Biomarkers	Result
Foley et al. (2012) [23]	Saliva and serum	Myocardial necrosis	TnI, CK-MB, and MYO	Elevated in both, but less dramatically in saliva. Significant correlation (*p* < 0.001) serum and saliva for matrix metalloproteinase-9, C-reactive protein, and myeloperoxidase observed.
Foley et al. (2012) [23]	Saliva and serum	Inflammation, tissue injury and remodeling	CRP, MMP-9, MPO	Elevated in both saliva and serum, but a greater MPO downward trend in saliva
Foley et al. (2012) [21]	Saliva and serum	Pre-existing CVD diseased patients who underwent an invasive cardiac procedure	CRP, TNF-α, sCD40L, IL-1β, IL-6, adiponectin, MMP-9, MPO, sICAM-1	Adiponectin, BNP, CK-MB, CRP, IL-6, MMP-9, MPO, MYO, TNFα, sCD40-L and sICAM-1 more elevated in serum; IL-1β higher in UWS TnI same levels
Miller et al. (2014) [4]	Saliva and serum	AMI	CRP, IL-6, IL-1β, MPO, sCD40L, TNF-α, Adip, sICAM-1, MMP-9	Tnl, BNP, CK-MB, MYO, CRP were all detected in statistically significant levels in the serum; Adip, sICAM, CRP were detected in statistically significant levels in saliva
Labat et al. (2013) [18]	Saliva and serum	Hypertension	Lysozyme	Saliva
Labat et al. (2013) [18]	Saliva and serum	IMT	CRP, MMP-9	CRP more in serum; MMP-9 more in saliva
Labat et al. (2013) [18]	Saliva and serum	Arterial stiffness	LTB4 and PGE2	Only saliva
Rathnayake et al. (2013) [1]	Saliva	Patients who had under gone heart surgery	MMP-8	Elevated
Rathnayake et al. [1]	Saliva	Hypertension	MMP-8, lysozyme	Elevated
Floriano et al. (2009) [15]	Serum and saliva	AMI patients within 48 h of chest pain onset		CRP, MMP-9, IL-1beta, sICAM-1, adiponectin, MCP-1, Gro-alpha, E-selectin, IL-18, ENA-78, sVCAM-1 were upregulated in saliva more than serum; MPO, MYO, CK-MB, TnI, BNP, sCD40-L TNF-α were upregulated more in serum than saliva; Fractalkine, IL-6, Adiponectin, MCP-1, Gro-alpha, E-selectin were downregulated.
Dizgah et al. (2013) [24]	Saliva and serum	12 and 24 h of onset of MI	cTnI	Elevated
Foo et al. (2012) [16]	Saliva and serum	Heart Failure patients	NT-proBNP	No correlation found between saliva and serum, though elevated in both.

**sVCAM-1** = Soluble vascularization cellular adhesion molecule-1, **TnI** = Troponin I, **Gro-α** = Growth related protein-alpha, **ENA-78** = Epithelial cell-derived neutrophil-activating peptide 78, **CK-MB** = Creatine kinase-myoglobin, **CRP** = C-reactive protein, **MMP-9** = Matrix metalloproteinase 9, **MPO** = Myeloperoxidase, **MYO** = Myoglobin, **TNF-α** = Tumor necrosis factor alpha, **sCD40L** = Soluble CD40 ligand, **IL-1β** = Interleukin 1 beta, **IL-6** = interleukin 6, **sICAM-1** = Soluble intracellular adhesion molecule, **Adip** = Adiponectin, **LTB4** = Leukotriene B4, **PGE2** = Prostaglandin E2, N-terminal, NT-**proBNP** = proB-type natriuretic peptide, **cTnI** = Cardiac specific troponin I, **BNP** = Brain natriuretic peptide, **MCP-1** = Monocyte chemoattractant protein-1, **UWS** = unstimulated whole salivasas.
